# Triglyceride glucose index for the detection of the severity of coronary artery disease in different glucose metabolic states in patients with coronary heart disease: a RCSCD-TCM study in China

**DOI:** 10.1186/s12933-022-01523-7

**Published:** 2022-06-06

**Authors:** Jinyu Su, Zhu Li, Mengnan Huang, Yang Wang, Tong Yang, Mei Ma, Tongyao Ni, Guangwei Pan, Ziqin Lai, Chunjie Li, Lin Li, Chunquan Yu

**Affiliations:** 1 Tianjin University of Traditional Chinese Medicine, 10 Poyanghu Road, West Area, Tuanbo New Town, Jinghai District, Tianjin, 301617 China; 2 Tianjin Chest Hospital, 261 Taierzhuang South Road, Jinan District, Tianjin, 300350 China

**Keywords:** Coronary heart disease, Coronary artery disease severity, Triglyceride glucose index, Glucose metabolic states

## Abstract

**Background:**

Triglyceride glucose (TyG) index is a new marker associated with atherosclerosis. This study aimed to assess the association between TyG index and the severity of coronary artery disease (CAD) in patients with coronary heart disease (CHD) and further explore the association between TyG index and CAD severity in different glucose metabolic states.

**Methods:**

This multi-centre retrospective study included 731 patients with CHD between January 1, 2014 and September 30, 2020 in China. All patients were stratified into groups based on the tertiles of TyG index (T1: 5.48 ≤ TyG index ≤ 7.17; T2: 7.18 ≤ TyG index ≤ 7.76; T3: 7.77 ≤ TyG index ≤ 10.82). The number of diseased vessels [single-vessel and multi-vessel CAD (≥ 50% stenosis in ≥ 2 large vessels)] represented the severity of CAD, which was measured using coronary angiography (CAG). Glucose metabolic states were defined by the American Diabetes Association as normal glucose regulation (NGR), prediabetes mellitus (Pre-DM), and diabetes mellitus (DM).

**Results:**

The baseline analysis results showed significant differences in the clinical and biological characteristics of CHD patients according to TyG index tertiles (*P* < 0.05 to < 0.001). Logistic regression analysis showed that the TyG index was significantly related to the risk of multi-vessel CAD (odds ratio [OR]: 1.715; 95% confidence interval [CI] 1.339–2.197; *P* < 0.001). The OR for multi-vessel CAD in TyG index T3 compared to that of T1 was 2.280 (95% CI 1.530–3.398; *P* < 0.001). Receiver operating characteristic (ROC) curve was generated to evaluate the accuracy of the TyG index in detecting the CAD severity, and the area under the curve (AUC) of the ROC plots was 0.601 (95% CI 0.559–0.643). The association between TyG index and multi-vessel CAD was significant in patients with DM, achieving the highest OR among the different glucose metabolic states (OR: 1.717; 95% CI 1.161–2.539; *P* < 0.05).

**Conclusion:**

TyG index was associated with CAD severity in patients with CHD, and an increased TyG index could identify patients with a high risk of multi-vessel CAD. There was an association between TyG index and CAD severity for the condition of DM.

**Supplementary Information:**

The online version contains supplementary material available at 10.1186/s12933-022-01523-7.

## Background

Coronary heart disease (CHD) is a chronic cardiovascular disease (CVD) caused by the hardening of the coronary arteries. The morbidity and mortality of CHD are on the rise due to aging, urbanization, obesity, and unhealthy lifestyle habits. Therefore, CHD is becoming a serious public health problem [1, 2]. Type 2 diabetes mellitus (T2DM) is widely considered a prevalent comorbidity that affects the progression of CHD and the treatment choices for these patients [3]. Patients with CHD with a combined abnormal glucose metabolic state have a higher risk of adverse cardiovascular events [4–6]. Specifically, T2DM increases the probability of developing CHD and the risk of death from CHD by at least two-fold [7, 8].

In patients with CHD, the severity of coronary artery disease (CAD) varies and is assessed by the patient’s clinical symptoms and ancillary investigations. Previous studies have found that 42.4% of individuals who experienced sudden cardiac deaths caused by CHD had no clinical symptoms; however, on autopsy, these individuals were found to have severe CAD and myocardial infarction [9, 10]. Coronary angiography (CAG) is a common and accurate imaging test known as the “gold standard” for diagnosing CAD in China. CAG may accurately detect the number and degree of arterial stenosis in patients. Based on CAG results, CHD is defined when there is lumen stenosis of ≥ 50% in at least one major coronary artery. The number of diseased vessels infers the severity of the CAD. However, many patients are unable to undergo CAG testing due to its high cost or invasive nature and, consequently, miss out on the right medical assessments. Therefore, it is necessary to develop a simple, valid, and widely applicable biomarker to detect the severity of CAD in patients with CHD.

Triglyceride glucose (TyG) index, a combination of fasting plasma glucose (FPG) and triglyceride (TG) calculations, is considered to be a representative biomarker of insulin resistance (IR) [11, 12]. Among the traditional risk factors, FPG and TG levels have been associated with an increased risk of T2DM [13, 14]. In addition, a large-scale Korean study indicated that the TyG index was associated with atherosclerotic CVD and has recently attracted considerable attention as a simple and low-cost biomarker of atherosclerosis [15]. TyG index is also associated with carotid arterial stenosis, and our previous study revealed that the relationship between TyG index and carotid artery plaques was significant in patients with CHD [16, 17]. Numerous studies have shown that an elevated TyG index is an independent risk factor for atherosclerosis [18–20]. Additionally, elevated TyG indexes play a potentially valuable role in the early recognition of individuals at high risk of CVD [21]. In the meantime, the severity of CAD plays a crucial role in the prognosis of CHD and the incidence of CVD. It is necessary to identify people at high risk of multi-vessel CAD among patients with CHD, especially those with abnormal glucose metabolic states are at an even higher risk. However, the ability of TyG index to detect CAD severity in patients with CHD has not been proven, and the influence of glucose metabolic states requires further study.

This study aimed to investigate the association between TyG index and CAD severity in patients with CHD and the effects of different glucose metabolic states on this association. Furthermore, this study determined the accuracy of the TyG index for detecting disease severity in patients with CHD.

## Methods

### Subjects

**Fig. 1:**
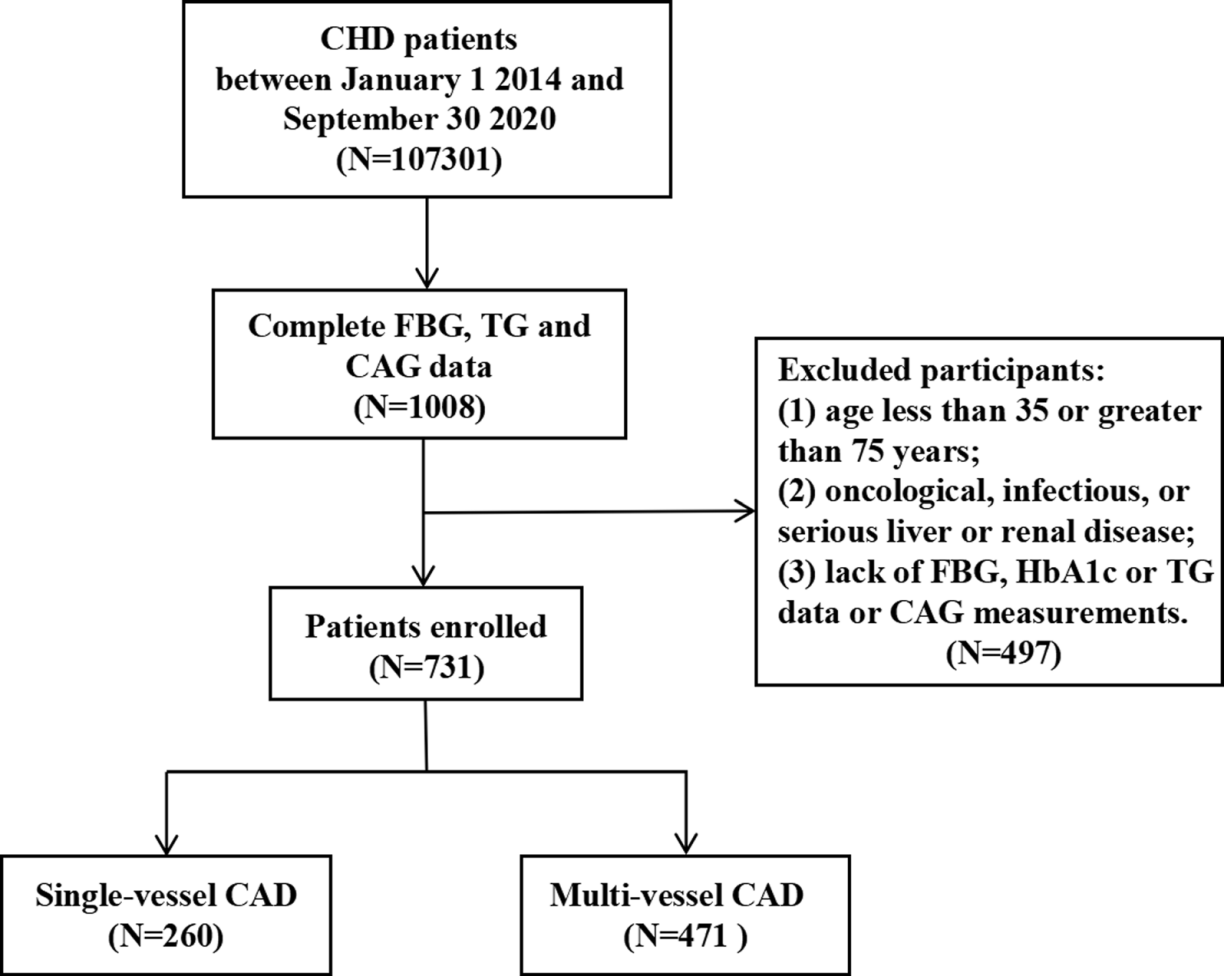
Flow chart of patient recruitment

This large-scale multi-center retrospective study population comprised 107,301 CHD patients hospitalized in Tianjin between January 1, 2014, and September 30, 2020. The following patients were excluded: (1) those younger than 35 years or older than 75 years; (2) those with tumors, infectious or severe liver or kidney disease; and (3) those who lacked data on glycated haemoglobin (HbA1c), FPG, TG, and CAG. Ultimately, 731 subjects were enrolled in the final analysis. The inclusion process is illustrated in Fig. 1. Approval for the study was obtained from the Ethics Committee of Tianjin University of Traditional Chinese Medicine (TJUTCM-EC20190008) and registered with the China Clinical Trials Registry (ChiCTR-1900024535) and with Clinical Trials. gov (NCT04026724).

### Data collection

The following data for the study analysis were acquired from medical records: clinical history, anthropometric data, blood analysis, and medical imaging data. Anthropometric data such as weight, height, body mass index (BMI), and blood pressure, and personal information such as age, sex, smoking history, and alcohol consumption history were recorded. The blood samples tested were fasting venous blood collected by specialist medical staff from all participants early in the morning. FPG, HbA1c, TG, total cholesterol (TC), high-density lipoprotein cholesterol (HDL-C), and low-density lipoprotein cholesterol (LDL-C) levels were measured using an automatic hematology analyzer. CAG was performed using a percutaneous femoral arteriography technique with an angiography machine with sufficient right and left anterior oblique views to diagnose all coronary artery lesions.

### Definitions

TyG index is derived arithmetically as follows: Ln[TG (mg/dL) × FPG (mg/dL)/2] [22]. BMI was calculated by dividing weight by square height and was expressed in kg/m^2^. CHD was defined as a lumen stenosis of ≥ 50% in at least one major coronary artery (left anterior descending, left circumflex, and right coronary arteries). The number of diseased vessels with ≥ 50% stenosis indicated the severity of CAD in patients with CHD. Patients with only one major coronary artery affected were considered to have single-vessel CAD, and those with two or more major coronary arteries affected were considered to have multi-vessel CAD. No other vascular lesions but stenosis of ≥ 50% in the left main coronary artery was regarded as two lesions. The participants were divided into the following two groups: single-vessel and multi-vessel CAD. Coronary stenoses (CS) was considered significant when one or more major branches of the coronary artery had ≥ 50% stenosis, moderate stenosis if 50–69%, and severe stenosis if ≥ 70%. According to the American Diabetes Association’s Standards for the Medical Treatment of Diabetes [23], diabetes mellitus (DM) is defined as FPG ≥ 7.0 mmol/L or HbA1c ≥ 6.5%, prediabetes mellitus (Pre-DM) is defined as 5.6 mmol/L ≤ FPG ≤ 6.9 mmol/L or 5.7% ≤ HbA1c ≤ 6.4%, and normal glucose regulation (NGR) is defined as FPG < 5.6 mmol/L or HbA1c < 5.7%.

### Statistical analysis

Continuous data are presented as mean ± standard deviation (SD) or median and interquartile range (IQR). Categorical variables are expressed as percentages. Differences between the groups were calculated using the χ^2^ test for categorical variables and the *t*-test, Mann–Whitney test or Kruskal–Wallis test for continuous variables. Odds ratios (OR) and 95% confidence intervals (CI) were calculated using logistic regression analysis to test the association between TyG index and CAD severity in patients with CHD. The area under the curve (AUC) and 95% CI of the receiver operating characteristic (ROC) curve were calculated to determine the accuracy of the TyG index in detecting CAD severity in patients with CHD. A *P-*value of < 0.05 was considered statistically significant. All statistical analyses were performed using the SPSS 24.0 (IBM Corp, New York, NY, USA).

## Results

### Clinical and biological characteristics

Of the 731 participants involved in this study, the average age was 63 (IQR, 58–68) years, 58.7% were male, and 64.4% had multi-vessel CAD. The patients were divided into three groups based on TyG index tertiles: T1 (5.48 ≤ TyG index ≤ 7.17), T2 (7.18 ≤ TyG index ≤ 7.76) and T3 (7.77 ≤ TyG index ≤ 10.82) in Table 1. Compared to participants in the lowest TyG index tertile (T1), participants in the highest tertile (T3) were associated with higher BMI, TC, TG, LDL-C, FPG, and HbA1c but lower HDL-C (*P* < 0.001). Furthermore, patients in T3 were more likely to have abnormal glucose metabolism, more diseased vessels, and more severe degree of CS than those in T1 (*P* < 0.001). The effect of age, sex, systolic blood pressure (SBP), diastolic blood pressure (DBP), and history of smoking and drinking did not show significant differences between the groups (*P* > 0.05).

**Table 1:** Clinical and biological characteristics according to TyG index tertiles

Characteristics	T1 (5.48–7.17) (n = 244)	T2 (7.18–7.76) (n = 244)	T3 (7.7–10.82) (n = 243)	*P-*value
Age (years)	64 (59, 69)	64 (58, 68)	63 (56, 67)	0.062
Sex (M/F)	1.32	1.57	1.38	0.634
SBP (mmHg)	140 (128, 156)	141 (129, 159)	140 (125, 156)	0.805
DBP (mmHg)	82 (76, 92)	83 (76, 90)	83 (76, 93)	0.750
BMI (kg/m^2^)	25.72 (23.65, 29.01)	26.67 (23.94, 29.33)	27.47 (24.61, 29.67)	< 0.001
Drinking	86 (35.2%)	90 (36.9%)	77 (31.7%)	0.468
Smoking	120 (49.2%)	124 (50.8%)	119 (50.0%)	0.905
TC (mg/dL)	4.27 (3.56, 5.04)	4.49 (3.91, 5.28)	5.04 (4.36, 6.00)	< 0.001
TG (mg/dL)	1.04 (0.84, 1.19)	1.58 (1.30, 1.96)	2.52 (1.87, 3.33)	< 0.001
HDL-C (mg/dL)	1.16 (0.97, 1.37)	1.05 (0.89, 1.22)	0.99 (0.80, 1.16)	< 0.001
LDL-C (mg/dL)	2.57 (1.99, 3.22)	2.74 (2.19, 3.31)	2.89 (2.30, 3.62)	< 0.001
FPG (mmol/L)	5.63 (5.09, 6.18)	6.31 (5.58, 7.60)	9.30 (7.01, 12.48)	< 0.001
HbA1c (%)	5.8 (5.5, 6.2)	6.1 (5.7, 6.8)	6.9 (6.0, 8.4)	< 0.001
TyG index	6.88 (6.65, 7.04)	7.42 (7.30, 7.60)	8.17 (7.94, 8.49)	< 0.001
Glucose metabolism state				
NGR	59 (24.2%)	26 (10.7%)	8 (3.3%)	< 0.001
Pre-DM	131 (53.7%)	97 (39.8%)	37 (15.2%)	
DM	54 (22.1%)	121 (50.8%)	198 (81.5%)	
Number of vessels with stenosis				
1	105 (43.0%)	94 (38.5%)	61 (25.1%)	< 0.001
≥ 2	139 (57.0%)	150 (61.5%)	182 (74.9%)	
Degree of coronary stenosis				
50% ≤ CS < 70%	76 (31.1%)	65 (26.6%)	45 (18.5%)	0.005
CS ≥ 70%	168 (68.9%)	179 (73.4%)	198 (81.5%)	

Data are presented as median (interquartile range) or number (proportion, %)

M, male; F, female; TyG, triglyceride glucose; SBP, systolic blood pressure; DBP, diastolic blood pressure; BMI, body mass index; FPG, fasting plasma glucose; HbA1c, glycated haemoglobin; TC, total cholesterol; TG, triglycerides; HDL-C, high-density lipoprotein cholesterol; LDL-C, low-density lipoprotein cholesterol; NGR, normal glucose regulation; Pre-DM, prediabetes mellitus; DM, diabetes mellitus; CS, coronary stenoses

The patients' baseline characteristics are listed in Table 2. The multi-vessel CAD group comprised more participants who were male and had abnormal glucose metabolism (*P* < 0.001). TyG index, FPG, TG, HbA1c, and LDL-C were significantly positively associated with multi-vessel CAD, while HDL-C was negatively associated (*P* < 0.05).

**Table 2:** Clinical and biological characteristics according to CAD severity

Characteristics	Total (n = 731)	Single-vessel CAD (n = 260)	Multi-vessel CAD (n = 471)	*P-*value
Age (years)	63 (58, 68)	63 (57, 68)	63 (58, 69)	0.361
Sex (M/F)	1.42	1.10	1.65	0.009
SBP (mmHg)	140 (127, 156)	140 (126, 154)	141 (127, 156)	0.225
DBP (mmHg)	83 (76, 91)	83 (76, 90)	83 (76, 91)	0.944
BMI (kg/m^2^)	26.67 ± 3.90	26.47 ± 3.75	26.86 ± 3.91	0.077
Drinking	253 (34.6%)	91 (35.0%)	162 (34.4%)	0.506
Smoking	363 (49.7%)	123 (47.3%)	240 (51.0%)	0.084
TC (mg/dL)	4.61 (3.87, 5.43)	4.45 (3.78, 5.21)	4.69 (3.92, 5.63)	0.018
TG (mg/dL)	1.51 (1.11, 2.17)	1.42 (1.05, 2.04)	1.57 (1.14, 2.21)	0.018
HDL-C (mg/dL)	1.05 (0.88, 1.24)	1.10 (0.92, 1.32)	1.03 (0.87, 1.21)	0.002
LDL-C (mg/dL)	2.72 (2.14, 3.39)	2.63 (2.02, 3.20)	2.80 (2.19, 3.50)	0.002
FPG (mmol/L)	6.44 (5.53, 8.66)	5.98 (5.28, 7.34)	6.71 (5.66, 9.40)	< 0.001
HbA1c (%)	6.1 (5.6, 7.0)	6.0 (5.5, 6.5)	6.2 (5.7, 7.3)	< 0.001
TyG index	7.42 (7.04, 7.94)	7.32 (6.93, 7.75)	7.52 (7.10, 8.06)	< 0.001
Glucose metabolism state				
NGR	93 (12.7%)	45 (17.3%)	48 (10.2%)	< 0.001
Pre-DM	265 (36.3%)	113 (43.5%)	152 (32.3%)	
DM	373 (51.0%)	102 (39.2%)	271 (57.5%)	
Drug therapy				
Antihypertensive	545 (74.6%)	196 (75.4%)	349 (74.1%)	0.461
Antilipidemic	561 (76.7%)	198 (76.2%)	363 (77.1%)	0.518
Antiplatelet	708 (96.9%)	246 (94.6%)	462 (98.1%)	0.010

Data are presented as mean ± standard deviation or median (interquartile range) or number (proportion, %)

M, male; F, female; TyG, triglyceride glucose; SBP, systolic blood pressure; DBP, diastolic blood pressure; BMI, body mass index; FPG, fasting plasma glucose; HbA1c, glycated haemoglobin; TC, total cholesterol; TG, triglycerides; HDL-C, high-density lipoprotein cholesterol; LDL-C, low-density lipoprotein cholesterol; NGR, normal glucose regulation; Pre-DM, prediabetes mellitus; DM, diabetes mellitus

### Association between TyG index and CAD severity

Binary logistic regressions were used to assess the associations between CAD severity and various risk factors. Multi-vessel CAD was used as the dependent variable (single-vessel CAD was used as a reference). There was no multicollinearity between the independent variables. Male, TC, LDL-C, FPG, and HbA1c were significantly associated with multi-vessel CAD (*P* < 0.05) when compared to patients with single-vessel CAD (Table 3). TyG index was positively associated with multi-vessel CAD in patients with higher ORs (OR: 1.715, 95% CI 1.349–2.179; *P* < 0.001).

**Table 3:** Associations between CAD severity and risk factors

Variables	Multi-vessel coronary artery disease		
	OR (95% CI)	β	*P-*value
Sex			
Female	Reference		
Male	0.666 (0.490–0.905)	− 0.406	0.009
Age	1.009 (0.989–1.029)	0.009	0.389
Smoking			
No	Reference		
Yes	1.307 (0.965–1.770)	0.267	0.084
Drinking			
No	Reference		
Yes	1.114 (0.810–1.531)	0.108	0.506
SBP	1.004 (0.997–1.012)	− 0.004	0.186
DBP	1.000 (0.988–1.012)	− 0.075	0.987
BMI	1.036 (0.996–1.077)	0.035	0.077
TC	1.182 (1.040–1.343)	0.237	0.010
TG	1.065 (0.946–1.199)	0.063	0.297
HDL-C	0.405 (0.233–0.704)	− 0.903	0.130
LDL-C	1.328 (1.121–1.573)	0.284	0.001
FPG	1.148 (1.084–1.217)	0.138	< 0.001
HbA1c	1.356 (1.191–1.544)	0.304	< 0.001
TyG index	1.715 (1.349–2.179)	0.539	< 0.001

Compared with single-vessel CAD

OR, odds ratios; CI, confidence interval; β, regression coefficient; TyG, triglyceride glucose; TC, total cholesterol; TG, triglycerides; HDL-C, high-density lipoprotein cholesterol; LDL-C, low-density lipoprotein cholesterol; FPG, fasting plasma glucose; HbA1c, glycated haemoglobin

As shown in Table 4, logistic regression models were constructed to show that the TyG index was significantly related to CAD severity before and after multivariate adjustment (*P* < 0.001). When the TyG index was analyzed as a continuous variable, it was significantly associated with multi-vessel CAD (OR: 1.715; 95% CI 1.339–2.197). When the TyG index served as a classified variable, the risk of multi-vessel CAD for patients in T3 was 2.280 times greater than the risk for patients in T1 (95% CI 1.530–3.398, *P* < 0.001) after adjusting for confounding factors. An elevated TyG index was significantly associated with an increased risk of multi-vessel CAD.

**Table 4:** Association between TyG index and CAD severity

Variables	Multi-vessel coronary artery disease					
	OR (95% CI)^a^	*P-*value	OR (95% CI)^b^	*P-*value	OR (95% CI)^c^	*P-*value
TyG index	1.715 (1.349–2.179)	< 0.001	1.764 (1.383–2.251)	< 0.001	1.715 (1.339–2.197)	< 0.001
T1	Reference		Reference		Reference	
T2	1.205 (0.840–1.730)	0.311	1.200 (0.833–1.728)	0.328	1.122 (0.774–1.627)	0.543
T3	2.254 (1.533–3.313)	< 0.001	2.357 (1.595–3.848)	< 0.001	2.280 (1.530–3.398)	< 0.001
*P-*trend		< 0.001		< 0.001		< 0.001

Compared with single-vessel CAD

T1: 5.48 ≤ TyG index ≤ 7.17; T2: 7.18 ≤ TyG index ≤ 7.76; T3: 7.77 ≤ TyG index ≤ 10.82

OR, odds ratios; CI, confidence interval; TyG, triglyceride glucose

^a^Model 1: unadjusted

^b^Model 2: adjusted for age and sex;

^c^Model 3: adjusted for age, sex, SBP, DBP, BMI, smoking, drinking, and antihypertensive, antilipidemic, and antiplatelet drug therapy

**Fig. 2:**
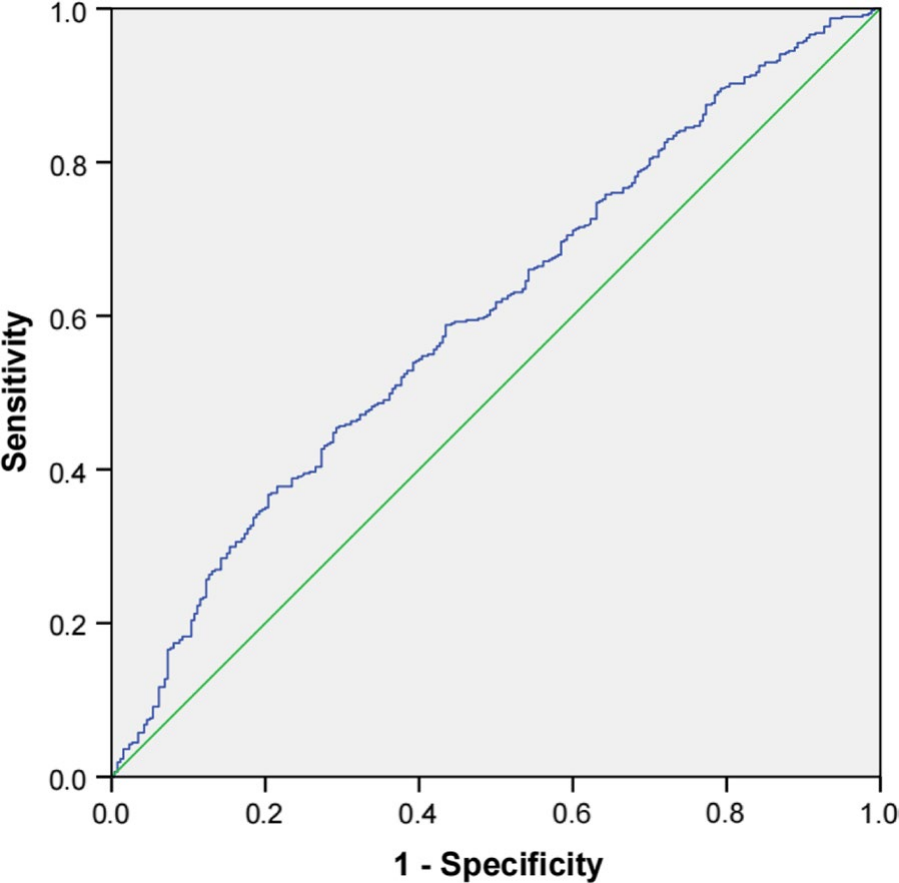
ROC curve for the use of TyG index in the detection of multi-vessel CAD

The ROC curve for multi-vessel CAD and TyG index is shown in Fig. 2. The results show that a TyG index of > 8.0 indicated that the number of affected coronary arteries was ≥ 2. The AUC for TyG index was 0.601 (95% CI 0.559–0.643; *P* < 0.001).

### Associations between TyG index and CAD severity in different glucose metabolism states

Binary logistic regressions were used to assess the impact of glucose metabolic states on the relationship between TyG index and CAD severity (Table 5). There was no statistically significant association between TyG index and the presence of multi-vessel CAD for NGR and Pre-DM (*P* > 0.05). However, there was a statistically significant association for DM, which had the highest observed OR value (OR: 1.717; 95% CI 1.161–2.539, *P* < 0.001). Taking T1 as a reference, T3 was significantly associated with a 2.105 times increased risk of multi-vessel CAD for patients with CHD and DM (OR: 2.105; 95% CI 1.069–4.144, *P* < 0.05).

**Table 5:** Associations between TyG index and CAD severity according to different glucose metabolism states

Glucose regulation state	Variables	Multi-vessel coronary artery disease					
		OR (95% CI)^a^	*P-*value	OR (95% CI)^b^	*P-*value	OR (95% CI)^c^	*P-*value
NGR	TyG index	1.828 (0.802–4.166)	0.151	1.747 (0.726–4.203)	0.213	1.797 (0.706–4.573)	0.219
	T1	Reference		Reference		Reference	
	T2	1.292 (0.512–3.257)	0.588	1.227 (0.458–3.287)	0.684	1.329 (0.467–3.783)	0.594
	T3	3.321 (0.619–17.820)	0.161	3.052 (0.529–17.608)	0.212	3.373 (0.545–25.628)	0.180
Pre-DM	TyG index	0.990 (0.623–1.574)	0.966	1.055 (0.658–1.691)	0.824	0.926 (0.565–1.517)	0.182
	T1	Reference		Reference		Reference	
	T2	0.918 (0.540–1.562)	0.753	0.945 (0.553–1.616)	0.837	0.868 (0.449–1.509)	0.759
	T3	0.825 (0.396–1.719)	0.608	0.905 (0.430–1.908)	0.794	0.791 (0.367–1.707)	0.616
DM	TyG index	1.646 (1.127–2.404)	0.010	1.721 (1.170–2.532)	0.006	1.717 (1.161–2.539)	0.007
	T1	Reference		Reference		Reference	
	T2	1.191 (0.610 -2.327)	0.609	1.192 (0.609–2.334)	0.608	1.030 (0.513–2.069)	0.933
	T3	2.185 (1.142–4.181)	0.018	2.273 (1.180–4.376)	0.014	2.105 (1.069–4.144)	0.031

Compared with single-vessel CAD

OR, odds ratios; CI, confidence interval; NGR, normal glucose regulation; Pre-DM, prediabetes mellitus; DM, diabetes mellitus

^a^Model 1: unadjusted

^b^Model 2: adjusted for age and sex;

^c^Model 3: adjusted for age, sex, SBP, DBP, BMI, smoking, drinking, and antihypertensive, antilipidemic, and antiplatelet drug therapy

## Discussion

This is the first study to reveal a significant association between TyG index and CAD severity in patients with CHD and assess this association according to different states of glucose metabolism. In the present study, the TyG index was significantly associated with CAD severity. Among the different states of glucose metabolism, the association between TyG index and multi-vessel CAD was significant in patients with DM.

In this retrospective cohort study, we observed that the prevalence of abnormalities in clinical and biological characteristics was higher than in the general population. The results showed that an elevated TyG index was also associated with severe dyslipidemia, progressively impaired glucose metabolic status, and coronary stenosis progression. This may be because our study was hospital-based, and participants had CHD with more risk factors. Epidemiological studies have identified multiple important risk factors responsible for the progression of CAD, such as IR, hypertension, hyperlipidemia, and obesity [24, 25]. Our results corroborated that patients with CHD have abnormal biochemical parameters and glucose metabolic statuses when experiencing progressive CAD.

Previous literature has suggested that the TyG index is related to the degree of coronary artery stenosis and could identify asymptomatic patients with T2DM who are at high risk of coronary artery stenosis [26]. In line with this study, our research supports this notion by more thoroughly demonstrating that the TyG index is associated with CAD severity in patients with CHD. TyG index has become a simple surrogate marker for IR, which is a fundamental clinical feature of severe metabolic syndrome and a marker for a collection of pathological conditions associated with systemic inflammation, endothelial dysfunction, oxidative stress, and prothrombotic states [27]. A 13-year follow-up study reported that metabolic syndrome was a critical CAD risk factor that leads to approximately twice the amount of CAD mortality [28]. Patients with IR result in the disappearance of the normal coordinated hypoglycemic response and the loss of, for example, inhibition of endogenous glucose production and lipolysis, cellular uptake of available blood glucose, and net glycogen synthesis [29]. During IR, TG stored in adipose tissue undergoes lipolysis, which produces fatty acids before raising blood glucose levels [30]. Abnormal blood glucose and lipid metabolism are risk factors that lead to poor CVD prognosis [31]. Therefore, IR is considered a pivotal risk factor for cardiometabolic diseases [32]. IR plays an essential role in the atherosclerotic process by increasing the risk of atherogenic damage, causing myocardium damage and atherosclerotic plaque generation [33]. TyG index performs better than the homeostasis model assessment of insulin resistance in predicting subclinical atherosclerosis [34, 35]. Consistent with the above literature, multi-vessel CAD was associated with an elevated TyG index in patients with CHD.

It is well known that multi-vessel CAD is a type of CHD with a high risk of adverse events and death and is a clinical indication for coronary artery bypass grafting. This study showed that an increased TyG index was significantly associated with the risk of multi-vessel CAD regardless of confounders, such as age, sex, SBP, DBP, history of smoking and alcohol consumption, and drug therapy, further emphasizing the strength of this association. Additionally, the study’s findings showed that the incidence of multi-vessel CAD was higher in patients with DM than in those with Pre-DM and NGR (72.7% > 57.4% > 51.6%) and that the TyG indexes of these patients were significantly related to multi-vessel CAD. Studies have indicated that DM presents with dyslipidemia which increases CHD risk [3, 36]. Compared to individuals with NGR and Pre-DM, patients with DM have a higher prevalence and severity of CAD [37]. DM may increase cardiovascular risk by accelerating the development of atherosclerosis [38, 39]. The pathogenesis of diabetic atherosclerosis involves hyperglycemia, dyslipidemia, changes in secretion of hormone other than insulin, and a proinflammatory state. Oxidative stress and inflammation interact in the development of abnormal glucose metabolic states to accelerate atherosclerosis [40].

IR is a critical pathophysiological pathway leading to DM and is present for an extended period before diabetes is diagnosed [41]. Previous studies have stated that the TyG index can indicate IR in individuals without diabetes [42]. However, we found no association between TyG index and CAD severity in patients with NGR or Pre-DM. In contrast to the theory above, we found that the TyG index has some limitations for detecting CAD in patients with CHD with NGR or Pre-DM. TyG index may only be optimal for detecting multi-vessel CAD in patients with DM and not for patients with NGR or Pre-DM. Further investigation is required to assess the exact parameters of this index in the detection of multi-vessel CAD in patients with different metabolic states.

## Strengths and limitations

A strength of this study is that we explored the association between TyG index and the severity of CAD in different glucose metabolic states, which to our knowledge, is the first to be conducted. In addition, the data were recorded and described by professional doctors, and the results were reconfirmed using sensitivity analysis. This may increase their reliability in determining whether the TyG index can provide a new basis for predicting and diagnosing CAD severity. This study also had some limitations. First, we could not determine the existence of a causal relationship due to the inherent limitations of retrospective studies. Second, the study population was relatively small. Third, the participants were inpatients; thus, there may be some limitations to the generalizability of the results to other populations.

## Conclusion

In conclusion, this study demonstrated that the TyG index was associated with the CAD severity in patients with CHD using a cohort dataset. An elevated TyG index could identify patients with CHD with a high risk of multi-vessel CAD. There was an association between TyG index and CAD severity among patients with DM, while no association was observed in patients without DM. Testing TyG index can be used as a risk management strategy for patients with CHD with different glucose metabolic states to assist in determining the severity of CAD and in making more appropriate healthcare decisions.

## Supplementary Information

**Additional file 1: Table S1.** Association between the TyG index and the number of vessels with stenosis ≥ 50%. **Table S2.** Association between the TyG index and degree of coronary stenoses.

## Data Availability

The datasets used and/or analyzed in the current study are available from the corresponding author upon reasonable request.

## Abbreviations

TyG: Triglyceride glucose
CAD: Coronary artery disease
CVD: Cardiovascular disease
CHD: Coronary heart disease
CS: Coronary stenoses
CAG: Coronary angiography
NGR: Normal glucose regulation
Pre-DM: Prediabetes mellitus
DM: Diabetes mellitus
T2DM: Type 2 diabetes mellitus
SBP: Systolic blood pressure
DBP: Diastolic blood pressure
BMI: Body mass index
FPG: Fasting plasma glucose
HbA1c: Glycated haemoglobin
TC: Total cholesterol
TG: Triglycerides
HDL-C: High-density lipoprotein cholesterol
LDL-C: Low-density lipoprotein cholesterol
IR: Insulin resistance
OR: Odds ratios
CI: Confdence interval
β: Regression coefficient
