# Supplementary material for: Triglyceride glucose index for the detection of the severity of coronary artery disease in different glucose metabolic states in patients with coronary heart disease: a RCSCD-TCM study in China
Source: Cardiovasc Diabetol. 2022 Jun 6;21:96. doi: 10.1186/s12933-022-01523-7 (PMC9169264; doi:10.1186/s12933-022-01523-7)
Supplement: Supplementary file 1 — Additional file 1: Table S1. Association between the TyG index and the number of vessels with stenosis ≥ 50%. Table S2. Association between the TyG index and degree of coronary stenoses. [file 12933_2022_1523_MOESM1_ESM.docx]

**Table S1. Association between the TyG index and the number of vessels with stenosis ≥ 50%**

| **Variables** | **1** | | **2** | | **3** | |
| --- | --- | --- | --- | --- | --- | --- |
|  | OR(95% CI) | *P* value | OR(95% CI) | *P* value | OR(95% CI) | *P* value |
| Model 1 | Ref | Ref | 1.508(1.156-1.968) | 0.002 | 2.025(1.526-2.686) | <0.001 |
| Model 2 | Ref | Ref | 1.535(1.172-2.011) | 0.002 | 2.125(1.591-2.838) | <0.001 |
| Model 3 | Ref | Ref | 1.514(1.146-1.999) | 0.003 | 2.125(1.572-2.871) | <0.001 |

OR, odds ratios; CI, confidence interval; Ref, Reference

Model 1: unadjusted

Model 2: adjusted for age and sex;

Model 3: adjusted for age, sex, SBP, DBP, BMI, smoking, drinking, and antihypertensive, antilipidemic, and antiplatelet drug therapy

Compared with 1 diseased vessel.

**Table S2. Association between the TyG index and degree of coronary stenoses**

| **Variables** | **CS ≥ 70%** | | | | | |
| --- | --- | --- | --- | --- | --- | --- |
|  | OR(95% CI)^a^ | *P* value | OR(95% CI)^b^ | *P* value | OR(95% CI)^c^ | *P* value |
| TyG index | 1.702（1.305-2.219） | <0.001 | 1.710（1.306-2.240） | <0.001 | 1.680（1.276-2.212） | <0.001 |
| T1 | Ref |  | Ref |  | Ref |  |
| T2 | 1.246(0.841-1.844) | 0.272 | 1.222(0.821-1.818) | 0.322 | 1.171(0.779-1.759) | 0.449 |
| T3 | 1.990(1.305-3.036) | <0.001 | 2.037(1.327-3.127) | 0.001 | 2.034(1.312-3.154) | 0.002 |
| *P*-trend |  | <0.001 |  | <0.001 |  | <0.001 |

OR, odds ratios; CI, confidence interval; Ref, Reference; CS, Coronary stenoses; TyG, triglyceride glucose;T1: 5.48 ≤ TyG index ≤ 7.17; T2: 7.18 ≤ TyG ≤ 7.76; T3: 7.77 ≤ TyG ≤ 10.82

^a^Model 1: unadjusted

^b^Model 2: adjusted for age and sex;

^c^Model 3: adjusted for age, sex, SBP, DBP, BMI, smoking, drinking, and antihypertensive, antilipidemic, and antiplatelet drug therapy

Compared with 50%≤CS<70%.
